# Biomaterials: Foreign Bodies or Tuners for the Immune Response?

**DOI:** 10.3390/ijms20030636

**Published:** 2019-02-01

**Authors:** Erminia Mariani, Gina Lisignoli, Rosa Maria Borzì, Lia Pulsatelli

**Affiliations:** 1 Laboratorio di Immunoreumatologia e rigenerazione tissutale, IRCCS Istituto Ortopedico Rizzoli, Via di Barbiano 1/10, 40136 Bologna, Italy; gina.lisignoli@ior.it (G.L.); rosamaria.borzì@ior.it (R.M.B.); lia.pulsatelli@ior.it (L.P.); 2 Dipartimento di Scienze Mediche e Chirurgiche, University of Bologna, Via Massarenti 9, 40138 Bologna, Italy

**Keywords:** biomaterials, immune response, macrophages, scaffold, foreign body reaction, extra-cellular matrix

## Abstract

The perspectives of regenerative medicine are still severely hampered by the host response to biomaterial implantation, despite the robustness of technologies that hold the promise to recover the functionality of damaged organs and tissues. In this scenario, the cellular and molecular events that decide on implant success and tissue regeneration are played at the interface between the foreign body and the host inflammation, determined by innate and adaptive immune responses. To avoid adverse events, rather than the use of inert scaffolds, current state of the art points to the use of immunomodulatory biomaterials and their knowledge-based use to reduce neutrophil activation, and optimize M1 to M2 macrophage polarization, Th1 to Th2 lymphocyte switch, and Treg induction. Despite the fact that the field is still evolving and much remains to be accomplished, recent research breakthroughs have provided a broader insight on the correct choice of biomaterial physicochemical modifications to tune the reaction of the host immune system to implanted biomaterial and to favor integration and healing.

## 1. Introduction

Biomaterials play a central role in a wide variety of healthcare issues and have fostered great improvements in different biomedical fields, such as tissue engineering, medical implants, drug delivery, and immunotherapies [1,2,3,4,5]. This wide applicative potential relies on the ability of these materials to provide biocompatible supports (i.e., scaffolds, devices), to encapsulate and protect biological active products (i.e., cells, chemicals, and proteins), and to allow easy modification of chemical and physicochemical properties [5,6,7,8,9,10]. Biomaterials include a broad range of compounds that widely differ in function and structural features, ranging from naturally occurring biological macromolecules to fully synthetic coatings. 

However, one common property of biomaterials is the induction of adverse immune reactions resulting in excessive inflammation, impairment of healing, fibrotic encapsulation, tissue destruction, or even isolation and rejection of medical devices. 

A more in depth understanding of the material/biological environment interplay is greatly needed, in order to develop strategies and solutions to overcome side effects in the use of these devices, which still represent an important challenge in the biomedical field. 

In this review, we detail the different cellular and molecular events characterizing biomaterial-immune system interactions. Then, we discuss how the immune response can be tuned by biomaterial properties (such as surface chemistry and topography) and by decellularized extracellular matrix. Finally, we highlight how the specific features of the different biomaterials could be exploited to control the inflammatory-immune response to implanted biomaterials and to promote tissue regeneration. 

## 2. Immune System—Biomaterial Interplay

The immune response is a biological network in charge of protecting the host from foreign threats and maintaining homeostasis.

The human immune system comprises two arms: the innate immune system, which elicits a non-specific inflammatory response following the immediate recognition of foreign material, and the adaptive immune system, which performs highly specific antigen responses and develops a long-term memory. Each part includes different cell populations: polymorphonuclear cells, mononuclear phagocyte cells (dendritic cells—DCs, monocytes, and macrophages) and lymphocytes (natural killer cells, gamma delta T-cells, and innate lymphoid cells) belong to the innate system, whereas B and T lymphocytes belong to the adaptive one [11]. The development of an appropriate and effective immune response requires close, coordinated, and carefully controlled crosstalk between the two systems, by means of soluble factors and cellular subsets.

Implantation of a biomaterial induces a host reaction to the implant that determines the outcome of the integration and the biological performance of the implant. Degradation products released by devices (tissue engineered scaffolds, orthopedic implants, biomedical devices) and the resulting surface changes of the degrading biomaterials activate the immune system [12]. The interplay between the host immune system and the biomaterial depends on the tissue surrounding the implant, which will drive the tissue-specific innate defenses and the following induction of adaptive immune responses. In fact, it is becoming more apparent that macrophages resident in tissues or recruited from other sites play distinct roles in the healing process likewise implantation of the same material into different sites elicits distinct responses [13].

**Figure 1:**
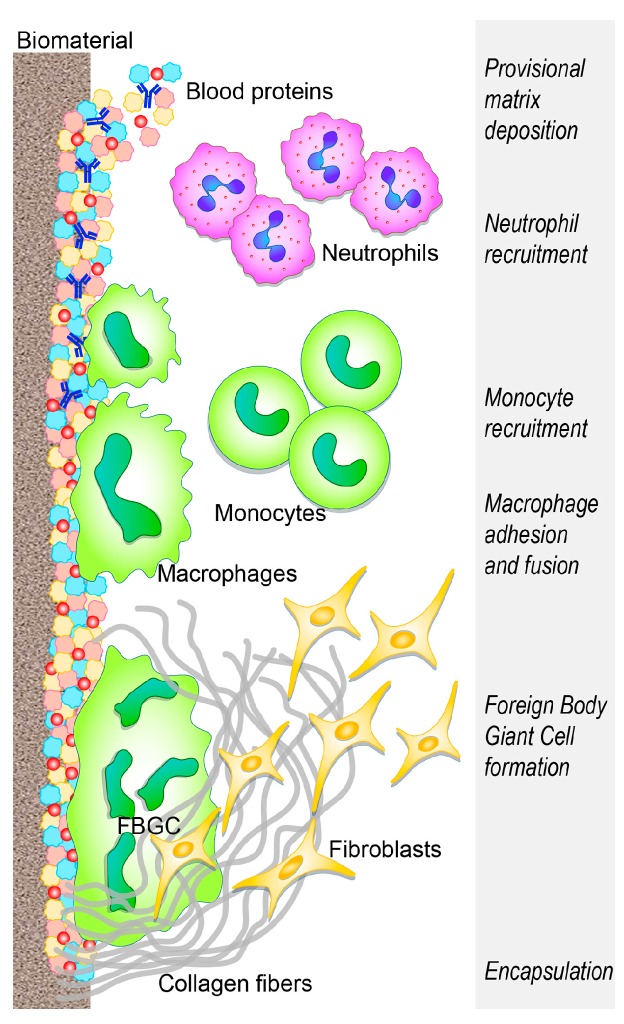
Innate immune response to biomaterials: the development of the foreign body reaction. The main cellular players in the biomaterial-immune system interaction are represented. The main events, from the initial biomaterial implantation to fibrous encapsulation, are schematically described.

The benefit and functionality of the implanted biomaterial can be weakened by the development of an acute sterile inflammatory reaction (foreign body reaction—FBR) superimposing tissue vascularization and remodeling, and ending with a fibrotic encapsulation that prevents further interplay between the biomaterial and the host tissue (Figure 1) [14,15,16] (extensively reviewed by [1,17,18,19]). 

Even if biomaterial implants have the ability to induce a FBR according to context specific features, the clinical manifestations widely differ for gravity and for the resulting implant outcome [6,12,19,20]. However, FBR is only one possible outcome of biomaterial implantation and the possibility to modulate this response is the key for successful implantation. 

Within a few seconds from implant placement, blood from the damaged vessels surrounds the biomaterial, thus beginning the interaction with the implant. Within minutes, host plasma components, including proteins (albumin, fibrinogen, fibronectin, vitronectin, and gammaglobulins), lipids, sugars, and ions, are rapidly and spontaneously adsorbed on the implant surface [6,21]. Various characteristics of the biomaterial surface (such as energy, chemistry, topography, and roughness) influence the type, the amount, the composition, and the conformation changes of adsorbed molecules and further recruitment and adhesion of tissue-derived, inflammatory, vascular, and stromal cells, in the following hours/days [22,23,24]. These characteristics are crucial determinants of the tissue reaction to such implants [6,25].

The blood exudate also contains platelets and other components of the coagulation cascade, and the resulting clot formation defines the provisional matrix around the biomaterial [26,27], leading to further platelet activation and aggregation [28,29] and to fibrinogen cleavage into fibrin by means of thrombin [30]. In addition, complement proteins, activated upon contact with the biomaterial, synergistically support platelet adhesion and activation [31,32,33] and the recruitment and adhesion of additional immune cells [34,35], attracted by the local bulk of pro-inflammatory cytokines, chemokines, and growth factors [12,36]. Following the formation of the provisional matrix, the acute and chronic inflammatory responses follow each other and the intensity of these responses is influenced by the wideness of the implantation damage [12] and also by the implanted biomaterial. Extra-cellular matrix (ECM) adhesion proteins, including fibronectin and vitronectin, have also been reported to adhere to biomaterial surfaces [37], and are crucial in modulating the inflammatory reaction to biomaterials, whereas fibrinogen and complement are mainly involved in the activation of the cellular component of inflammation. Fibronectin and vitronectin respectively enhance cell adhesion [38,39,40], promote macrophage fusion, and participate in the FBR chronic phase [41,42,43].

Cell adhesion to protein-coated biomaterials and the subsequent cell activation are mediated by integrin signals [7,44,45]. In addition, leukocytes undergo activation by means of the same systems of surface receptors that detect foreign microorganisms. In particular, some pattern recognition receptors (PRRs) (usually interacting with pathogen-associated molecular patterns—PAMPs, found on microorganisms and primarily expressed on macrophages and dendritic cells) [46,47] are also able to sense dangerous situations. They induce immune responses driven by molecules within the family of damage-associated molecular patterns (DAMPs). Other danger signals participating in leukocyte activation are Alarmins (which include heat shock proteins, high mobility group box 1, ATP—adenosine triphosphate, uric acid). They are endogenous equivalents of PAMPs, and they are similarly recognized by macrophages and DCs, through PRRs (here acting as scavenger receptors), Toll-like receptors (TLR), and C-type lectin [48,49,50].

Activated neutrophils, recruited from the peripheral blood by chemoattractant factors (released from host activated platelets, endothelial cells, and injured tissue cells), adhere to the implantation site (by means of β2 integrins) and attempt to destroy/degrade the biomaterial through phagocytosis, proteolytic enzymes, and reactive oxygen species (ROS) released by cytoplasmic granules [51,52,53,54]. In addition, neutrophils release neutrophil extracellular traps (NETs) [55], a “sticky network” of granular proteins, neutrophil elastase, chromatin DNA, and histones [56], usually involved in trapping pathogens and preventing infection spread [55]. Altered release of neutrophil extracellular traps is involved in sustaining the fibrotic tissue response, leading to the excessive production of a dense fibrotic matrix [57]. The undue production of NETS prevents integration between the tissue and the biomaterial and degrades neutrophil-produced cytokines and chemokines that regulate the healing process [57,58]. This impairs the healing response and the potential for tissue regeneration, and promotes fibrotic encapsulation [59]. Furthermore, NETs release from neutrophils, unable to phagocytose a harmful stimulus [60], may be considered similar to the formation of foreign body giant cells by the fusion of frustrated macrophages [61].

Upon activation, neutrophils synthesize a significant amount of immune-regulatory signals [62]: CXCL(CX chemokine ligand)8 (the most prominent chemokine), whose primary targets are neutrophils themselves, CCL (C chemokine ligand) 2 and CCL4, both potent chemoattractant and activation factors for monocytes, macrophages, immature DCs, and lymphocytes [63]. The progressive increase of these chemokines fosters monocyte infiltration and suppresses that of neutrophils, which, lacking further activating signals, undergo apoptosis and progressively disappear from the implantation site [12]. Concurrently, circulating monocytes respond to chemoattractant (such as CCL2, CCL3, and CCL4) and bind fibrinogen in the biomaterial provisional matrix, thereby undergoing activation [43,64,65] and differentiation into the classically activated or “M1” macrophages [42,66,67,68]. These cells have been classified according to their ability to secrete pro-inflammatory cytokines, (such as interleukin (IL)-1β, IL-6, tumor necrosis factor-TNFα), chemokines [66,69], and enzymes.

Adherent macrophages foster the invasion of additional inflammatory cells by secreting chemokines (CCL2, CCL4, CXCL8) [69] and also attempt to degrade the biomaterial by releasing ROS and degrading enzymes [70,71], before undergoing “frustrated” phagocytosis (since the biomaterial is too large to be internalized), ultimately resulting in an increased release of cytokines [72,73]. Similar to the wound healing process [74], adherent macrophages eventually shift to the “M2” phenotype [75], secreting anti-inflammatory cytokines (such as IL-10) presenting a reduced degradative capacity and achieving tissue remodeling activity, also inducing the migration and proliferation of fibroblasts toward effective tissue regeneration [76]. The overlapping events of the phenotypic M1 to M2 switch (reviewed by [74]), as well as the mechanisms of frustrated phagocytosis, result in macrophage membrane fusion to form a foreign body giant cell (FBGC, an hallmark of chronic inflammation) on the biomaterial surface. This reflects an attempt to increase their phagocytic functionality, to attach and degrade large implants [71,77]. FBGC can adhere to the surface of the biomaterial for a long time, thus forming a barrier between the tissue and device, and eventually ending with implant deterioration and/or loss. Therefore, macrophage plasticity allows their adaptation to immune-regulatory, host defense, tissue repair roles in response to the implant properties. The formation of FBGCs is often a signature component of biomaterial-induced FBR and is fostered through the activation of mast cells, basophils, and T helper (Th) cells that secrete IL-4 and IL-13 shown to enhance macrophage fusion on biomaterials [78,79,80]. Mast cells are consistently reported at the site of implantation [81,82,83], where they degranulate upon activation, releasing histamine (shown to play a role in directing neutrophils and monocytes to implanted biomaterials [82,84] and secreting pro- and anti-inflammatory cytokines, angiogenic, and pro-fibrotic factors (such as vascular endothelial growth factor—VEGF and transforming growth factor—TGF-β) [85,86]. 

During the chronic inflammatory phase, cytokines are mainly produced by directly or indirectly activated T lymphocytes, mainly CD (cluster of differentiation) 4 helper T cells and their Th1 and Th2 subsets. Their copious cytokine production widely modulates the pro/anti-inflammatory responses [78]. The correlation between the M1/M2 macrophage phenotype and the change in cytokine expression profile from Th1 to Th2 lymphocytes suggests that T lymphocytes are pivotal in promoting the resolution of inflammation and regeneration. With regards to this aspect, some evidence points out the importance of the early neutrophil immune responses for the later modulation of M2 macrophages and Th2 lymphocytes to functional healing [87,88,89]. T cell involvement in the FBR to non-phagocytosable implants has not been fully elucidated; however, T cells have been shown to attach to the biomaterial [90] and become activated through non-canonical pathways [91,92,93]. They also enhance macrophage adhesion and fusion into FBGCs through paracrine actions of secreted cytokines [78,94,95]. 

The concerted action of immune cells results in the release of pro-fibrogenic factors, such as platelet-derived growth factor (PDGF) [96], VEGF [97,98], and TGF-β [99,100], which recruit fibroblasts. In an attempt to repair the damaged tissue, activated fibroblasts deposit collagen (type I and III); however, excessive secretion results in the undesirable final outcome of fibrotic deposition of ECM (greater ratio of I/III is associated with a greater fibrotic tissue formation) [101], encapsulating the biomaterial [102,103] and compromising implant function [104,105]. The deposition of a new matrix is mainly carried out by fibroblasts and myofibroblasts, the prominent cellular components in the fibrotic reaction. Myofibroblasts (as suggested the “myo” prefix) share proteins of the smooth-muscle cells, whose contraction can concur to fibrotic scar formation [106].

With regards to the pro-regenerative mechanisms, M2 macrophages displaying an anti-inflammatory/anti-fibrotic phenotype contribute to regeneration through crosstalk with a subpopulation of T cells defined as regulatory (Tregs), which play an important role in tissue immune homeostasis. These cells can skew the local immune response from inflammation to a pro-regenerative tissue repair cascade, sustaining the anti-inflammatory/anti-fibrotic phenotype by the secretion of anti-inflammatory cytokines, such as IL-10. Furthermore, Tregs are able to improve healing quality by inducing a type 2 response, including anti-inflammatory macrophages. Following a T cells decrease, resident Treg levels remain elevated, probably because they display an epithelial growth factor receptor (EGF-R) [107,108] whose expression allows the growth factor amphiregulin secreted by mast cells to maintain Tregs at the damaged site [107]. Once present, Tregs proliferate and upregulate the secretion of amphiregulin, which may induce either cell proliferation [109] or differentiation and is necessary for regeneration [110,111,112]. Tregs may also enhance the regenerative capacity of endogenous stem/progenitor cells through the secretion of growth factors.

In addition to the above reported cells and mechanisms, whose involvement in the response to biomaterials is well-described even if not exhaustively explained, other cells have been suggested to play roles in the timely resolution of inflammation and successful regeneration process.

For example, dendritic cells (DCs), similar to macrophages, phagocytize particles and process danger signals at the injury site. Although their exact role during tissue repair and regeneration is still not completely known [113], studies show that they play an important role in the tissue healing process [113,114]. DCs interact with T cells and B cells to initiate and shape the adaptive immune response, they have immuno-regulatory activities, and they influence the development of tolerogenic or anergic T cells, depending on their maturation stage, location, and cytokine environment [115]. Additionally, they induce the activation and growth of regulatory T cells (Treg) (reviewed in [115,116]). The interaction between dendritic cells and biomaterials was mainly studied in the presence of an immunogenic stimulus and the increased immune responses to co-delivered antigens [117] pointed at a novel “adjuvant” role of the biomaterial. However, this role assumes the ability of the biomaterial to activate DCs by direct contact between the material and the cells [118]. It is suggested that biomaterials prime dendritic cells through PRR signaling pathways [48] and depending on which PRR receptor is triggered, maturation or inhibition of DCs can be induced, contributing respectively to the lengthening of the immune response to biomaterials and the delay of wound healing or to the down-regulation of the inflammatory response [119]. 

Since multiple physio-pathological processes cannot be simply explained by the Th1 and Th2 cell paradigm, this led to the identification of a distinct T cell effector subset, referred to as Th17, the main IL-17 and IL-22 producing cells. Th17 cells, as well as IL-17 and IL-22, are basic components of the mucosal immune system and their alteration is closely linked to autoimmune and inflammatory diseases [120]. A recent report linked IL-17 mediated signaling to the differentiation of monocyte/macrophage populations to a pro-fibrotic phenotype [121]. However, IL-17 is redundantly produced by other immune cell populations, including γδ T cells [122] and innate lymphoid cells type 3 (ILC3) [123].

The γδ T cell subset has a prevalent surveillance role in native tissue [124] and has been widely reported as pro-regenerative, contrary to the αβ T cell subset that displays both pro- and anti-regenerative characteristics [124,125,126]. Few data show the beneficial role of the γδ T cell subset, as described in both mice and humans that do not effectively heal skin wounds in the absence of these cells [127,128]; however, little is known about their potential pro-regenerative contribution in the field of biomaterials. 

Another subset, the CD8 cytotoxic T cells (CTL) (responsible for detecting and killing cancer cells and viral infected targets), has been found to influence wound healing, as demonstrated by the improvement of its outcome following CD8 T cell depletion in rats [129] and by the negative impact on bone fractures, following a CD8 T cells increase in humans [130]. 

In addition to T cell depletion, few available evidence on the role of B cells in tissue healing seems to suggest that their depletion also represents a promising strategy to augment bone regeneration, since adaptive immune system deficient mice exhibit faster bone healing [131]. However, much is still to be discovered on the role of B cells in the repair and regeneration of various tissues.

Recently, innate lymphoid cells (ILCs), defined by the lack of expression of T or B cell receptors, have been subdivided into three classes (ILC1, 2, 3), characterized by their canonical transcription factors and cytokine expression [132]. These subsets somewhat mirror the expression of Th1, Th2, and Th17, respectively. ILC2, similar to Th2 cells, produces IL-4, IL-5, and IL-13 and like Th2 and M2 macrophages, is anti-inflammatory and provides a set of cell signaling, mediators and metabolites that are associated with wound healing. ILC2 also promotes CD4 T cell polarization towards Th2 cells via positive inhibition of Th1 [133,134,135]. Considering that the development of a pro-regenerative response to biomaterials requires type 2 cell populations [136] and that the crosstalk Th2/ILC2 is central to tissue pro-regenerative responses, ILC2 activity may be relevant with biomaterials.

The design of biomaterials targeted to tune the immune response to their benefit must therefore take into account the activation of immune cells and the mutual crosstalk between the different innate and adaptive cellular components [137].

## 3. Immunological Profile of Biomaterials

Biomaterials have been classified in many different ways, with the most immediate and general one referring to the chemical nature of the material itself. It is thus possible to distinguish metallic materials (ferrous and non-ferrous) and non-metallic materials (organic and inorganic). Among the organic materials, polymers (both synthetic and of natural origin) are of particular importance, whereas among the inorganic ones, ceramic materials must be mentioned [138]. 

Metal materials are mainly used in the manufacture of prostheses or implants for orthopedics and dentistry, as parts of composite implants. The most used metals are stainless steels, cobalt alloys, titanium and its alloys. However, metals have problems concerning biocompatibility in relationship to bone-metal interface processes and ion release, so metal-based implants must be treated to prevent the onset of inflammatory processes. Moreover, from the point of view of tissue engineering, the use of these materials is very limited.

The most used materials for tissue regeneration with particular attention to bone and cartilage regeneration are ceramics, synthetic polymers, and natural polymers [2,8]. 

Ceramic materials (such as glass, aluminum oxide, zirconium oxide, calcium phosphate) are mostly applied as orthopedic and dental implants and are also widely used as bone cavity fillers. They are characterized by a high hardness, high temperature resistance, low elasticity, and high fragility. They display an excellent biocompatibility thanks to the chemical and structural formulation analogous to the native bone tissue [139]. 

Composite materials, consisting of two or more types of materials, are each identifiable for the presence of interfaces between the components. The peculiarity of composite materials is that, being the combination of several components, they can provide better overall properties than individual constituents.

Polymeric materials represent 45% of the biomaterials used in the biomedical field. Generally, they are macromolecules formed by the more or less regular repetition of basic monomeric units. They allow customized architectures with a controllable degradation speed to be realized. However, their composition can lead to inflammatory phenomena and their hydrolytic degradation can release carbon dioxide (CO_2_) with a consequent decrease in the local pH, thus damaging the cells and inducing surrounding tissue necrosis [140]. Synthetic polymers have been demonstrated to be promising biomaterials for tissue engineering, due to their biomechanical and biodegradability properties. Natural polymer resemblance to the native ECM makes these scaffolds highly biocompatible. The main characteristics and the immunological profiles of both synthetic and natural polymers are summarized in Table 1.

Among the synthetic polymers, poly(caprolactone) (PCL), poly(lactic acid) (PLA), poly(glycolic acid) (PGA), and poly(lactic-co-glycolic acid) (PLGA) are currently the most popular for the creation of scaffolds [143,144]. 

PCL is a bioresorbable polyester material, considered to be both a soft and hard tissue compatible material [145]. PCL is elastic in comparison to other polyesters and is used in various forms, such as films, fibers, and microparticles. PCL also has poor cellular adhesion properties on its own, without some form of functionalization. 

PLA has a wide variety of applications, e.g., prosthetics, vascular grafts, skin regeneration, scaffolds, and bone screws [143]. To overcome the possible inflammatory reactions induced by crystalline fragments released during degradation by the lactic acid L isomer form, PLA can be obtained as a blend of L and D monomeric isoforms, thus lacking high crystallinity and improving degradation [143]. 

PGA degrades almost completely within a few months under an in-vivo condition. It is a good material used for bone tissue engineering applications.

PLGA is among the most widely used synthetic polyesters for scaffold formation in tissue engineering applications [143,146]. The major advantages of PLGA are the biodegradability, adaptability, and customization of different types of formulations or surface modifications. Since lactic acid, the basic component of the polymer, is also a product of cell metabolism, good ‘biocompatible’ properties are attributed to PLGA and its degradation-byproducts. However, one major limitation is the acidity of the degradation products that, if produced in large quantities, can hamper rapid and efficient metabolization by the body: increasing data point out that the frustrated degradation process and/or derived by-products may be able to induce an immune response [147]. The ratio among monomers can influence the tissue response to copolymers like PLGA.

Natural polymers include materials made from many polysaccharides (such as agarose, alginate, chitosan, hyaluronan, glycosaminoglycans) and proteins (such as collagen, fibrin, silk).

Agarose and alginate are natural polysaccharides typically obtained from red and brown seaweed, respectively. Agarose resemblance to the extracellular matrix results in attractive features for tissue engineering. It is an excellent candidate for controlled/localized drug delivery and a suitable biomaterial for cell-encapsulation because of the high water uptake capability [148]. In the biomedical field, alginates are used in medical textiles, hemostatic material, and wound dressing, for controlled drug release, for cells encapsulation, as scaffolds in ligament and tendon tissue engineering, and in preparing molds in dentistry. They are used as a stabilizer in solution and for the dispersion of solid substances by pharmaceutical industries [149]. 

Chitin is a naturally occurring mucopolysaccharide present in the exoskeleton of crustaceans, insects, and fungal cell walls. Chitosan (deacetylated form of chitin) has been reported for its various important pharmacological properties. It is mainly utilized as an excipient for tablets, as a controlled release dosage form, as gels, as an absorption enhancer, for drug delivery, in wound healing products, and in developing micro/nanoparticles. Its role in tissue engineering and regenerative medicine is also well-documented [150]. 

Glycosaminoglycans (GAGs) are long chains formed by repeated units of disaccharides. These unbranched carbohydrates have a fundamental role in life, being responsible for the coordination of manifold fundamental processes for tissue development and homeostasis, such as biophysical characteristics, cell signaling, and assembly of the extracellular matrix. GAGs are included in various biomaterials used for tissue engineering, drug delivery, and regenerative medicine [151].

Hyaluronic acid (HA) is the only non-sulfated GAG [152]. HA is a major component of the extracellular matrix (ECM) and plays an important role in regulating tissue injury, accelerating tissue repair, and controlling disease outcomes. Improved therapeutic efficacy has been achieved through partial modification and formation of HA-based biomaterials, including HA-scaffolds, sponge-like hydrogels, anti-adhesive sheets, cultured dermal substitutes, thin membranes, and dermal matrix grafts.

Collagen is the most abundant structural protein and is among the most frequently used naturally-derived polymers. It forms a highly organized, three-dimensional architecture and can carry any component due to its network-like structural nature. Collagen is used for different biomedical applications: as biomaterial for tissue engineering and bone substitute and for eye implants, as well as a matrix for drug, gene, and protein delivery and in sponges for burns/wounds [153].

Fibrin is an integral part of the clotting cascade and is formed by polymerization of the soluble plasma protein fibrinogen. Due to its role in hemostasis and tissue repair, fibrin has been used extensively as a tissue sealant. It is particularly interesting because, being fibrinogen and thrombin, obtained from autologous blood, the choice of fibrin allows the production of the patient’s own scaffold, providing the starting support for cell adhesion, migration, growth, and differentiation [154].

Silks are proteins contained in the glands of arthropods. Recently, silk from Bombyx mori (silkworm) larvae cocoons has been extensively discussed. It is a natural protein polymer containing 70–80% fibroin (core protein) and 20–30% sericin (adhesive protein) as major constituents. Silk fibroin (SF) has become a popular biomaterial due to its biocompatibility, robust mechanical performance, adjustable degradation, easy processing, and availability. Due to their paramount achievement, biomaterials based on silk fibroin may be used for different purposes, such as for the regeneration of vascular tissue, bone scaffolds, drug delivery, and dressing of skin wounds [155,156].

As shown in Table 1, well-studied biomaterials (such as alginate, agarose, chitosan, hyaluronic acid, PLGA) that are not or low antigenic in appearance, can differently crosstalk with the immune system, thereby eliciting complex and not fully understood immunomodulating properties [157]. Despite attempts to reduce the immunological response to the material by tuning its chemical and topographical surface characteristics, the synthetic materials trigger the classical foreign body reaction. The need for preventive co-drug treatment of various synthetic polymer surfaces (even if poorly adhesive for proteins), to avoid potential thrombotic or complement mediated reactions, demonstrated that the perfect material is not yet available [158]. 

Conversely, naturally-derived polymers, together with decellularized tissues, decellularized cellular constructs, and coatings, do not induce the typical foreign body reaction, but a positive innate immune remodeling reaction, associated with an adaptive immune response [158]. A better knowledge of scaffold behavior will have an impact on the control of both natural and synthetic constructs, giving a degree of engineerability to the natural polymers and developing tailored immune modulating constructs using synthetic ones [157,159,160].

Even if silk is less immunogenic, as a natural polymer, it induces a macrophage response [161], mostly mediated by the sericin protein, an adhesive matrix coating that traps fibroin. A controlled release of cytokines from a coated silk polymer induced and reversed M1/M2 macrophage polarization [162]. Moreover, a localized, short-term release from silk biomaterials, of either interferon (IFN)-γ or IL-4, shifted macrophage polarization from M1 to M2 [162]. Large silk biomaterials do not induce peripheral T-cell activation, probably due to markers downregulating the responsiveness of T lymphocytes [163]. 

Silk can participate in the composition of hydrogels, which are an important class of polymers characterized by highly hydrophilic properties. 

Hydrogels are obtained from natural sources, including ECM proteins (collagen, fibrin, gelatins) and polysaccharides (glycosaminoglycans—GAG, dextran, alginate and chitosan), as well as from synthetic sources that are poly(vinyl alcohol)-based. Hydrogels from native tissues can be used as scaffolds for tissue engineering, by sacrificing the original architecture but keeping the native biochemical cues. They can deliver biomolecules and mirror the ECM structure, thus supporting adhesion, proliferation, and differentiation of the embedded cells [138]. High molecular weight hyaluronic acid [164] and chitosan [165] have intrinsic anti-inflammatory properties due to their radical oxygen species-scavenging properties. However, the majority of biomaterials require loading or functionalization.

Natural polymers such as collagen and fibrin, susceptible to enzymatic degradation, are ideal for releasing immune modulating molecules. On the contrary, synthetic biomaterials potentially subjected to degradation and responsible for a possible immune response when implanted, need a close control over degradation and kinetics of molecule release [166]. 

## 4. Tunable Properties of Biomaterials

Increasing knowledge on the host responses to biomaterials, the processes of healing, and the potential of biomaterials to modulate immune cells, clearly points out two main developing strategies: the need for testing biomaterial effects on both innate and adaptive immune responses and the design of biomaterials capable of tuning appropriate immune responses at implantation sites [167,168]. Current strategies of such biomaterial design start from the surface properties, shown to be central to the immune response, and intervene either passively on the physicochemical characteristics or actively by incorporating molecules or coating matrices. 

### 4.1. Surface Chemistry: Hydrophobicity, Chemical Moieties, and Charge Characteristics

Biomaterial surface interaction with the adsorbed proteins is crucial for the reaction to the implants, as previously described [6,25]. Different methods of altering surface chemistry have been tested for creating poorly adhesive surfaces, in order to control the amount, composition, and conformational changes of bound proteins [169]. 

Biomaterial hydrophobicity plays an intrinsic immunogenicity [9,170]. The immune system has evolved to recognize hydrophobic portions of biological molecules as universal damage-associated molecular patterns, thus triggering PRRs and leading to elimination [170]. Therefore, hydrophobicity and hydrophilicity (surface wettability) are important factors affecting protein adsorption. The average unfolding of a protein molecule [171] and the overall spreading [172] are larger on hydrophobic surfaces than on hydrophilic surfaces, on which proteins preserve their native-state secondary structure and show little, if any, adsorption [173,174]. 

Gold nanoparticles functionalized with increasing hydrophobic chemical groups show a correlation between the degree of hydrophobicity and gene expression of pro-inflammatory cytokines (TNF-α, IFN-γ) by bystander cells [9]. Similarly, silica particle surfaces functionalized with amino acids of increasing hydrophobicity up-regulate IL-1β secretion from DCs and IFNγ secretion from T cells primed by these DCs [175]. Furthermore, hydrophobic particles undergo increased phagocytosis and clearance by the reticuloendothelial system [159].

The lower hydrophilicity of the pristine titanium surfaces induces a higher secretion of several pro-inflammatory cytokines (such as TNF-α, IL-1β, and CCL2) compared to heparin/fibronectin immobilized titanium surfaces [176]. On the contrary, hydrophilic modification of sand-blasted, acid-etched (SLA) surfaces [78] down-regulates the gene expression of several human macrophage pro-inflammatory cytokine genes (TNF, IL-1α, and β; CCL1, 3, 19, and 20; CXCL1 and 8; and IL-1 receptor type 1) and the secretion of the corresponding proteins [177].

With regards to macrophage and lymphocyte adhesion and activation, hydrophilic/anionic surfaces promote higher levels of macrophages and FBGC-adherent lymphocytes. Hydrophilic/neutral surfaces are selective for CD4 T lymphocyte interactions, while hydrophobic surfaces are selective for CD8 T lymphocyte interactions [178]. 

To counteract the immunogenic effects of hydrophobic surfaces, hydrophilic molecules such as polyethylene oxide (PEO) and polyethylene glycol (PEG) are often added to tissue engineering scaffolds and used as monolayer coatings to reduce surface protein absorption and as delivery vehicles to increase hydrophilicity [179,180,181]. However, an opposite approach has recently been suggested. It privileges the preservation of native three-dimensional protein conformation (in fact, protein unfolding or misfolding and presentation of cryptic bioactive sites can trigger adverse reactions) rather than the exclusion of the implant from all interactions with the surrounding tissue [182].

Another important surface characteristic is represented by chemical groups. The most commonly explored chemical moieties are amino (-NH_2_), carboxyl (-COOH), hydroxyl (-OH), and methyl (-CH_3_) groups [183]. Some of the main chemical characteristics and their involvement in the immunological response are summarized in Table 2.

Briefly, amino and hydroxyl groups induce the highest infiltration of inflammatory cells in vivo [185,190,194] and the thicker fibrotic capsules around the functionalized implant [190,195]. Amino terminated nanorods were also described to induce an anti-inflammatory M2 phenotype, whereas carboxyl ones shifted macrophages to an inflammatory M1 phenotype [187]. However, negatively charged carboxylated surfaces smooth inflammatory reactions [186,190,196] and trigger alterations in the migratory behavior and function of circulating macrophages, thus abrogating macrophage-mediated inflammatory activity and promoting regulatory T cell phenotypes [189]. 

Cell differentiation and focal adhesions [7,197] were maximally induced by hydroxyl moieties, followed by amino and carboxyl groups, and finally by methyl groups that were also able to induce a low myogenic differentiation [7,192]. 

Contradictory results have been reported on the effects of implants functionalized with hydrophobic, neutral -CH_3_ moieties; on the thickness of the fibrotic capsule; on the recruitment of inflammatory cells [186]; and on cellular adhesion [185,197,198]. 

Both cell differentiation and focal adhesions are regulated by integrin binding [44] and their different engagement mirrors conformational changes induced by adsorbed proteins. In fact, accessibility to fibronectin domains, integrin binding, and cell adhesion follow the same chemical moiety order [191,193]. 

The complement C3b component can covalently link the -OH group [199,200]. Considering that Complement receptor 3 (CR3)(CD11b/CD18) is expressed on leukocytes (mostly neutrophils) and macrophages, this offers an explanation for the increased accumulation of CD11b+ cells at the implant site [196]. 

The negative charges of both -COOH groups and the cell membrane, as well as the tighter adhesion of proteins (such as albumin and fibrinogen) [201,202,203] to hydrophobic surfaces [204], may account for some of the above results. However, they are not enough to foresee the behavior of bound proteins on these surfaces.

It was shown that proteins adsorbed to -COOH or -NH_2_ hydrophilic surfaces (negatively and positively charged, respectively) undergo greater conformational changes [203]. These data suggest that the amount of adsorbed proteins and their refolding degree are influenced by surface functional moieties rather than by the hydrophobicity degree. On the contrary, proteins were more prone to retain their native structure on -OH hydrophilic surfaces. 

The surface charge of a biomaterial represents another important player in the modulation of the immune responses. Particles with positively charged surfaces lead to activation of the inflammasome (a multi-protein intracellular complex that activates a highly pro-inflammatory signaling cascade of the innate immune system) to greater extents than negatively charged particles [187,205]. Particles with a negative surface charge can inhibit the immune function by preventing uptake of the materials by antigen-presenting cells, thus completely abolishing antibody and T cell responses [206]. Zwitterionic motifs show the ability to activate monocytes and DCs, (stimulated to up-modulate MHC (major histocompatibility complex) class II and costimulatory molecules and to produce cytokines) [207], as well as to induce less inflammatory response and a thinner fibrotic capsule, and to shift macrophages to an M2 phenotype [23].

However, contradictory results are found in the literature given that surface charges have also been described to do the opposite: the negative ones inducing the highest levels of activation and the positive ones (shown above to activate pro-inflammatory responses) inducing lower levels of IL-1β [175].

Alginate and hyaluronic acid are naturally derived, negatively charged biomaterials, that have been highly studied as scaffold materials. The opposite results derived from these studies, on the effect of the negatively charged surface on immune responses, highlight the need for more detailed studies, to fully understand how to take advantage of surface charge and material formulation in supporting regenerative outcomes.

Overall results have revealed that protein binding kinetics and conformations on implant surfaces are dependent on surface chemistry. Hence, the initial cell response is triggered by the adsorbed protein, rather than by the surface itself. The pattern in which adhesion proteins and other bioactive molecules adsorb elicits cellular reactions that are specific to the underlying material physicochemical properties. If on one hand, these aspects point out the difficulty in decoupling related properties, on the other hand, they highlight opportunities to modulate immune cell phenotypes by altering the hydrophobicity, charge, or surface chemical functional groups of materials used for tissue engineering constructs.

### 4.2. Topography: Size, Shape, and Surface Texture

Medical devices display intrinsic topographical characteristics either suitably introduced or resulting from the manufacturing process. Particle deposition, self-assembled monolayers, soft photolithography, blasting, acid etching, and polymer expansion [208,209,210] are examples of the numerous and different techniques available for modifying material topography. These methods give rise to different size geometries (nano, micron scale), surface protrusions (pillars, posts, gratings, ridges), or dentations (pits and dots) [98,211,212,213]. 

Topographical characteristics have been identified as important regulators of cellular and physiological processes [188], such as cell adhesion [214,215,216], density spreading and motility [217,218,219,220], proliferation and differentiation [219,221], macrophage fusion [98], and cytokine secretion [222,223].

As described previously, the pattern of adsorbed proteins regulates many phenomena at the nano-bio interface. Fine tuning of the adsorbed protein activity can be achieved by topographical changes at the nanometer scale, reflected by conformational changes of the adsorbed protein [224]. A gold nanoparticle surface (58 nm) is shown to increase serum IgG antibody adsorption by 70%; whereas the C3c complement fraction is decreased by 50% [225].

The increase of nanoscale roughness (from 15 nm to 30 nm) induces a binding affinity decrease of a panel of proteins (< or = 90%) and a relevant increase in adsorbed proteins (> or = 500%) [226]. 

Further studies have described the effects of nanotopography on the adsorption and modified conformation of fibrinogen [227] that increases with increasing root-mean-square roughness (from 2.0 to 32.9 nm). This is probably due to a change in the geometrical arrangement of the fibrinogen molecules on the surface, and to the adsorption of the cell-binding protein fibronectin [228], which shows a decrease in the thickness of the adsorbed fibronectin layer with decreasing bulk protein concentration. This potentially accounts for differences in cell adhesion and activation on flat versus rough surfaces. 

These data point out that the surface nanostructure and nanometric scale represent the relevant morphological characteristics regulating the protein adsorption process and that nanostructures are parameters that must be taken into account in the biomaterial design.

Different nanoscale topographies have also been explored for their interaction with cells [229]. However, larger surface patterns, ranging between 10 and 100 μm, are usually utilized to directly modulate cells [230]. Some of the effects induced on cells by different particle sizes are described in Table 3.

Various data are available for titanium implants for orthopedic and dental applications. Titanium, even if more biocompatible than other metals, induces remarkably strong inflammatory responses which represent important aspects of implant failure. The decrease of the immune response to titanium can be obtained by modifying titanium surfaces with nano/micro-structures or with chemical processes changing the surface roughness.

Other studies have demonstrated that cells can “bend” patterned pillars (likely due to filopodia contraction) [237] and a recent observation indicates that nanostructures could penetrate fibroblasts, likely due to failed phagocytosis, resulting in cell thinning and membrane rupture [238].

These examples collectively indicate that initial cellular activation can be modulated by nanoscale surface topography alone and highlight how the multitude of techniques and topographies can differentially affect cell responses. 

In addition to the size, the shape of a biomaterial is also an important parameter affecting the interaction with the immune cells, as indicated by some representative results reported in Table 4.

Overall, these studies reveal that the inflammasome can be modulated by simply altering the particle shape. Since tissue engineering can use biological or synthetic polymers, enabling a variety of shapes, the knowledge of how this parameter promotes or diminishes inflammasome activation could allow biocompatible and tolerated constructs to be produced. However, since these effects occur with some material shapes and not with others, more studies are necessary to fully understand the biology of this behavior. 

Recent interesting experimental approaches have used polymeric particles to obtain artificial antigen presenting cells (aAPCs) [243,245,246]. Spherical nanoparticles and various ellipsoidal particles (obtained by mechanical stretch of the spherical ones) were coupled with antigen and co-stimulatory molecules needed for bio-mimicking the correct presentation to T cells and their following activation and proliferation.

These studies demonstrate that spherical aAPCs are phagocytosed more quickly and at higher levels compared to ellipsoidal aAPCs, whereas ellipsoidal aAPCs present a greater biodistribution and a lasting presence in the bloodstream when injected intravenously in mice, thus allowing a better interaction with T lymphocytes, confirmed by their increased proliferation [246]. 

An optimal T lymphocyte proliferative response is obtained when aAPCs are stretched 2–2.5 fold, pointing out the importance of the stretching degree in supporting contact length and the resulting T cell-biomimicking aAPC interactions [245,246].

Titanium dioxide nanotubes, derived by titanium surface anodization, lead to a significant modification of the lipopolysaccharide-induced macrophage inflammatory response, thereby reducing the gene expression of cytokines and chemokines, protein synthesis, the development of FBGCs, and the release of nitric oxide (NO) [247].

A reduced density of macrophages is observed after 24 h culture on nano-textured and nano-tubular anodized titanium samples as a function of anodization voltage increase (10, 15, and 20 V), compared with conventional unmodified samples [248]. 

Bio-anodized, acid-etched, and machined titanium surfaces (Ti) do not influence macrophage viability and do not induce a macrophage cytokine release (IL1-β, TNF-α and TGF-β1) significantly different compared to the Ti surfaces. Furthermore, the Ti surface characteristics do not induce a typical Th1 or Th2 cytokine profile, suggesting that titanium surfaces are inert to monocytes/macrophages and do not change the characteristics of the cell response [249].

The use of porous materials has been investigated for several decades and has been integrated into areas of dentistry and orthopedics (dental and bone/joint implants) [250]. 

Although the ideal pore size for osteoblast functionality in implants for bone engineering is still disputed [251], pores ranging within 20–1500 µm [252] have been investigated for cell migration capacity, spreading, proliferation, cartilage and bone formation [253], and angiogenesis [254,255,256]. Many reports indicate optimal pore sizes and validate the model prediction. Others, at constant macroporous characteristics, point out that it is the material processing that influences the biological outcome [257]. 

Pore size diameters of 300–400 µm represent the optimal dimension for an effective bone formation in porous hydroxyapatite. In fact, straight tunnel structures with diameters of 350 µm allow the direct bone formation, whereas in tunnels with diameters ranging between 90 and 120 µm, cartilage formation precedes bone appearance [257].

The need for carrier geometries able to induce the development of vascular structures represents a further parameter to consider in designing systems for the effective reconstruction of joints and bones. Hydroxyapatite structural characteristics (pore size, geometry, continuity, and straightness) can be exploited for other biomaterials for regenerative medicine applications. In general, the porous nature of these implants is ideal because they allow for tissue integration, vascularization, and the transport of nutrients [250,256]. They are therefore suitable for the fabrication of large engineered constructs. As for the effect on macrophage polarity, porous versus nonporous poly(2-hydroxyethyl methacrylate- methacrylic acid) hydrogels induced iNOS, thus indicating that the biomaterial activates pro-inflammatory pathways. Macrophage mannose receptor positive cells increased significantly at porous implants (suggesting a shift to an M2 phenotype), concomitantly with improved neovascularization for implants with pores >20 μm [258]. A similar study [75] disclosed a positive correlation of increasing pore size with the expression of Arginase 1 (Arg, M2 marker), along with a negative correlation with the expression of inducible nitric oxide synthase (iNOS, M1 marker).

Dealing with expanded polymers characterized by increasing average intranodal distances, the largest distance of 4.4 µm induced an early pro-inflammatory activation of macrophages in vitro, characterized by high levels of IL-1β and TNF-α, together with the increased gene expression of chemokines, leading to the recruitment of monocytes and neutrophils. However, in a mouse model, the same intranodal distance led to a thinner, less organized, and less dense capsule surrounding the implant [211]. 

Porosity and pore size must also be considered from the perspective of the equilibrium between the scaffold porosity and the structural solidity needed for the implant, to ensure that its strength is not compromised [251]. If on one hand, increased porosity and expanded conformation of constructs influence macrophage function, promoting pro-regenerative environments, on the other hand, changes in scaffold structure may negatively affect mechanical strength. This aspect is particularly important for implants designed to replace anatomical tissue with structural functions, such as bone, for which the mechanical strength of the scaffold is essential. Thus, while scaffold shape and porosity can be handled to switch on inflammation or repair by modulating macrophage phenotype and foreign body cell formation, it is also necessary to better understand the interaction between these immunological outcomes and material properties [75,255].

Last but not least, topographical effects should also be addressed. Parallel line gratings of width ranging between 250 nm–2 µm did not induce a macrophage response distinctive of different grating sizes. On the contrary, different grating topographies were able to modify the macrophage response on every polymer surface, independently of surface chemical properties. Cellular morphology and cytokine production were affected in vitro, whereas cellular adhesion was affected in vivo, particularly on a larger size topography compared to planar controls [98].

Polymeric fibers modified to exhibit different shapes and assembled in scaffolds formed from either random or aligned fibers influenced macrophage behavior. Macrophages were able to penetrate into scaffolds comprised of randomly arranged fibers with expanded thickness (3 or 10 mm), implanted into rat subcutaneous tissue, whereas scaffolds formed from aligned fibers and expanded to a 3 mm thickness supported greater macrophage infiltration and a lower number of giant cells, likely due to the gap distance between the fibers [255]. Overall, published outcomes are often contradictory and difficult to compare due to disparity amongst various surface topographies. This points out the importance of using cell types appropriate for a given implant purpose in order to identify the optimal properties to achieve the desired response in vivo.

## 5. Immune-Interactive Strategies

For several decades, the design of biomaterials has been specially dedicated to the development of “passive” biomaterials, with the aim of limiting immune adverse reactions. Emphasis on enhancing tissue repair by downregulating an unwanted host inflammatory response to implants has led to the identification of strategies to hide implant surfaces such as immune-isolating coatings to passively prevent/reduce protein adhesion to the implanted biomaterial surface and the resulting leukocyte activation or hydrogels to isolate implants from immune cells and thus to limit the inflammatory response [259]. However, it has now become clear that the immune system is fundamental in orchestrating and defining the nature of the repair process [260,261]. Indeed, the inflammatory response is not an adverse reaction but a crucial gateway in tissue repair and regeneration [262] and allowing specific biological responses is beneficial for both biomaterial integration and performance [105]. 

The link between the immune response and repair is complex and the current challenge is the development of biomaterials and delivery systems able to modulate the immune system as a way of stimulating the repair of tissues and organs [263]. Accordingly, the concept of ideal biomaterial is moving from ‘‘immune-evasive” aiming at decreasing host responses to ‘‘immune-interactive” triggering desired immunological responses, therefore enabling biomaterial integration and subsequent tissue repair [1,19,264,265]. The numerous studies attempting to modulate biomaterial-immune system interaction by tuning the surface chemical properties and/or changing the topographical characteristics of biomaterials are mostly focused on macrophages, as previously reported. In addition, different strategies, such as the incorporation of bioactive molecules (adhesion sites, drugs, cytokines, growth factors, or pro-resolution mediators either alone or combined) [266], have provided rather interesting results. 

The following examples will focus on structures/molecules that are characteristic of the immune response, but are not exhaustive of the entire range of opportunities available for modulating interactions between the implanted biomaterial and the receiving host.

### 5.1. Immune Modulation by Decellularized ECM

The ECM is the non-cellular milieu spread within all tissues and organs that supplies both the fundamental physical framework for the cellular components and fundamental biochemical and biomechanical signals that regulate morphogenesis, differentiation, and homeostasis of the tissues.

Naturally derived scaffolds such as decellularized extracellular matrices are historically used as frames for reconstruction, for delivering cells and biological factors, and for controlled molecule release [267]. Although the development of biological scaffolds to deliver cells and factors remains an active area, the alternative strategy of using the scaffold to induce therapeutic immune responses or intentional shifts in the immune phenotype is emerging. Data from preclinical studies aiming at the development of ECM scaffolds for therapeutic purposes indicate the pivotal contribution of immune system modulation [268]; however, the basic mechanisms are not yet fully clarified.

Procedures of ECM decellularization collectively remove the tissue immunogenic components, reducing (but not eliminating) the antigen load and retaining only the native architecture. The response to a decellularized implant likely depends on many factors, including the tissue origin, the implant site, and the decellularization process, which influences the degree of immunogenicity of the remaining cell remnants. The structural properties of these natural scaffolds favor the organization and the functional recovery of the tissue by influencing numerous cellular processes that create a pro-regenerative environment and support the host infiltrating cells [269,270]. Indeed, among other properties, decellularized ECM has shown the ability to shift macrophage polarization towards either an M1 or M2 phenotype, thus modulating the wound immune microenvironment.

In agreement, the transplantation of acellular scaffolds has been generally connected to an M2-like response with less scarring and greater constructive remodeling capacities than cellular scaffolds [271] and it has been recently shown that scaffolds obtained from tissue ECM elicit a strong Th2 pro-regenerative immune environment, in turn enhancing M2 macrophage polarization via an IL-4-dependent pathway [136]. 

Collectively, these findings evidence that the induction of a Th2 environment is an important component of immune-interactive scaffolds in tissue engineering applications. However, the type of immune response induced by a decellularized ECM scaffold highly depends on the tissue from which the ECM is obtained. Since macrophages recognize denatured [272] and strain-damaged [273] collagen, different decellularization procedures may have different effects on macrophages and significantly bias immune activation, depending on the changes they induce in ECM components. Even if the exact underlying mechanism is still not fully clarified [271], it was recently suggested that biologically active microvesicles (MBVs) bound to ECM could be partially responsible for the scaffold dependent effects [274] by means of miRNAs present within MBVs. Although some MBVs miRNA are conserved across different sources, a significant amount are tissue-specific, thus miRNA specificity could be partially responsible for the different effects induced by decellularized scaffolds, depending on tissue origin. Indeed, the comparison of the macrophage response after exposure to ECM from different tissue sources shows very heterogeneous behaviors [275,276]: small intestine submucosa (SIS), urinary bladder, brain, esophageal, and colonic extracellular matrices induced an M2 phenotype similar to the one obtained by control macrophages incubated with IL-4. In contrast, dermal ECM induced an M1 phenotype with increased iNOS expression, while no shift was observed in macrophages treated with liver or skeletal muscle derived ECM.

In addition, since ECM proteins are highly conserved across species, xenografts are usually well-tolerated [269], thus diminishing the risk of undesired inflammatory responses which could interfere with the homeostasis of the immune environment. Interestingly, the capability to modulate the inflammatory response by means of macrophage polarization confers a higher tolerability to xenografts of acellular ECM compared to autologous grafts, in some cases. In different experimental models, decellularized xenogeneic tissues have extensively been shown to provide a better healing response, characterized by a reduced M1 macrophage presence and by shifts to an M2 phenotype, compared to autologous cellular material, as determined by immunohistological evaluations [277,278,279]. Moreover, decellularized ECM also represents an interesting carrier for the delivery of molecules. For instance, the sequential delivery of IFN-γ and IL-4 from decellularized bones switched the macrophage phenotype from M1(IFN-γ) to M2(IL-4) and increased vascularization of the bone scaffolds subcutaneously implanted in mice [280].

Another important characteristic of decellularized ECM is the mechanical properties, which depend on the source and the processing of the tissue. Compounds such as 1-ethyl-3-(3-dimethylaminopropyl) carbodiimide (EDC) and aldehydes are often used as cross-linkers to strengthen tissues and prevent degradation [281]. However, if on the one hand, the crosslinking supplies mechanical stability, on the other hand, the lack of degradation prevents implant remodeling by macrophages and other cells, and prevents replacing by native tissue, thus favoring a stronger FBR [282], further sustained by residual cross-linker macrophage toxicity and inflammation [283]. Even if complete decellularization is important for avoiding an inflammatory response [284], few studies have directly compared the effects of different decellularization protocols on immune activation. Moreover, a recent study of decellularized ECMs from different organs has used high-throughput screening techniques to define scaffold components. Quantitative analysis of tissue-specific responses (such as matrix production, cellular adhesion and growth, culture-dependent modification of morphology) has been found to correlate with tissue proteomics. A network analysis identified several proteins linked to cell function. For example, ECM glycoproteins, but not collagens, have been shown to affect macrophage activity. In particular, cartilage oligomeric matrix proteins and matrilin downregulate M1 function, whereas S100AB induces M2 activity [285]. The biochemical complexity of decellularized matrices is still poorly understood, so a better characterization of the active components of ECM will improve scaffold reproducibility. 

### 5.2. Immunomodulation by Pro-Inflammatory Molecules

Since the inflammatory response is the starting point of the tissue healing program, the use of pro-inflammatory molecules, including danger signals, to treat tissue damage, has been considered. Different studies highlight that targeting specific TLR pathways can force a desired response. Indeed, the HSP (heat shock protein)-70 endogenous agonist of TLR2/TLR4 [286] up-regulated macrophage-mediated phagocytosis [287], thus aiding healing; macrophage-activating lipopeptide-2, a TLR2/6 agonist, increased the vascularization of porous polyethylene without causing any local or systemic side effects [288] and CpG (cytosine-phosphorothioate-guanine) oligodeoxynucleotides designed to trigger human immune cells via TLR9 promoted skin repair [289].

Prostaglandin E2 (PGE2) belonging to a family of pro-inflammatory lipid molecules [290] has been involved in both pro- and anti-regenerative functions and it has also been shown to inhibit proliferation and skew the immune response to Th2 [290] by inhibiting IL-12 [291], IFN-γ, and IL-2 [292] secretion by human lymphocytes. While being beneficial for tissue healing, PGE2 administration requires multiple doses and presents significant side effects [293,294]. Therefore, the biomaterial-mediated local delivery of PGE2 would be better than repeated systemic administrations, as demonstrated in a mouse model [295]. A further improvement has been obtained by using an agonist specifically binding one of the four PGE2 receptors and slowly released via biomaterial [294,296].

Furthermore, the inflammatory and pro-angiogenic CXCL-12 chemokine has been shown to play an important role in the tissue repair process [297], in particular for its ability to mobilize progenitor cells [298] expressing the CXCR (CX chemokine receptor) 4 cognate receptor [299,300]. The usefulness of biomaterials delivering CXCL-12 has been demonstrated in different tissues, such as tendons [301], cardiac muscle [302], skin [303], and liver models [298].

### 5.3. Immunomodulation by Anti-Inflammatory Molecules

The use of anti-inflammatory factors represents another way to obtain immunomodulatory biomaterials.

Cytokines can be locally delivered either by immobilization into the biomaterial (such as hydrogels) or by nucleic-acid-based strategies that allow prolonged cytokine synthesis and release by the in situ implanted cells [304]. The hydrogel inclusion of TGF-β or IL-10 has been shown to be effective in suppressing the maturation of dendritic cells [305]. The sequential controlled delivery of IFN-γ and IL-4 from scaffolds or double hydrogel layers promoted the transition of M1 to M2 macrophages [280,306]. Besides, for delivering anti-inflammatory agents, polymeric hydrogels can also be designed for sequestering pro-inflammatory signals, as described for TNF-α and CCL2 [307,308]. The inability of the direct delivery system to sustain clinically relevant concentrations of cytokines for a long period of time has fostered the use of gene delivery-based systems which allow a desired concentration of the target cytokine to be maintained in the long term.

Cytokines such as IL-4 and IL-10 are fundamental for correct tissue repair and regeneration, because of their role in M1 to M2 switching [309]. For regenerative applications, IL-10 has been mostly delivered using plasmid DNA and virus vectors [310], or antibodies neutralizing the pro-inflammatory signals [311]. A decrease of the inflammatory response was obtained following scaffold implantation using a gene-therapy approach consisting of the localized delivery of IL-10 [264]. Both IL-4 delivery means (as a protein conjugated to a scaffold [280] or via injectable hydrogel [312]) were effective for inducing/increasing M2 macrophage polarization and tissue repair in a rat model. Delivery of IL-4 and IL-13 via biomaterials has also been extensively explored, with M2-skewed macrophage phenotypes having been observed [280,313]. Novel hydrogel-based gene delivery strategies have also been explored for the release of antisense oligodeoxynucleotides to downregulate local endogenous pro-inflammatory signals at the wound site [314,315].

Overall, it has been shown that introducing IL-4 or analogous anti-inflammatory cytokines into scaffolds may prevent undesired side effects of implanted materials [316] and the delivery may support M2 macrophage driven regeneration.

TNF-α has been shown to positively regulate tissue repair and regeneration in some situations; however, its excessive concentration can be detrimental to the healing process. Thus, strategies aimed at inhibiting the expression of this cytokine have been suggested to decrease the pro-inflammatory macrophage effect. The local administration of common painkillers such as aspirin [317], ibuprofen [318], and pentoxifylline [319] has shown interesting results in TNF-α reduction. The anti-TNF-α molecule conveyed by hyaluronic acid, taking advantage of the specific receptor (CD44) on macrophages, provides the signal directly to the cytokine producing cell [320]. On the other side, taking advantage of the tissue repair characteristics, pre-stimulation of mesenchymal stem cells (MSCs) with TNF-α has been shown to increase their engraftment to myocardial infarct [321] and its administration has been described to enable the mobilization of MSCs into damaged tissues [322].

TGF-β1, a further interesting factor, is required for the early stages of tissue repair [323], although this molecule can exert either inflammatory or anti-inflammatory properties, depending on the cell type it signals. For example, while TGF-β1 can inhibit the activity and proliferation of lymphocytes, at the same time, it can induce regulatory T cells [324]. Nevertheless, TGF-β1 also widely contributes to scar formation [323]. TGF-β3, one of the three isoforms of the cytokine, can be exploited to accelerate regeneration and avoid scarring [323], as demonstrated by prophylactic administration in human studies [325]. The design of a suitable delivery system for the β3 isoform may therefore exploit its anti-fibrotic properties in humans.

An additional molecule IL-33 [326], acting as both a cytokine and a nuclear factor, has been linked to fibrosis through the actions of leukocyte recruitment and modulation of ECM genes [327,328]. Upon secretion, IL-33 has been found to be a chemoattractant for Th2 cells [328], as well as an inducer of the secretion of IL-13 [329,330], an important cytokine involved in FBGC formation, by acting directly on Th2 cells via a constitutively expressed ST (suppression of tumorigenicity) 2 receptor [329,331]. The emerging links between IL-33 and fibrotic disorders [332,333] might suggest that the release of IL-33 at the biomaterial implantation site may induce collagen deposition. Further studies may qualify IL-33 as an additional candidate for a therapeutic blockade to reduce the FBR [334].

Glucocorticoids are potent immunosuppressor drugs of the immune responses [335]. They inhibit inflammatory mediator synthesis (including cytokines, chemokines, prostaglandins, leukotrienes, proteolytic enzymes, free oxygen radicals, and nitric oxide), concomitantly promoting anti-inflammatory cytokine release, Th2-immunity, and tolerance, while suppressing the Th1 response. The delivery of soluble pharmacological anti-inflammatory agents such as dexamethasone and heparin, incorporated onto implants through surface applied coatings, has shown reduced numbers of inflammatory cells and fibrotic capsule formation [336,337,338,339]. However, since the superficial coating only allows a short-term release of the selected factor limiting the length of inflammatory response modulation, the incorporation of nano- and microscale drug delivery systems into implants has been adopted, aimed at prolonging the immunomodulation time [310,340,341].

The combined delivery of glucocorticoids and anti-inflammatory cytokines (IL-6 and IL-10) can attenuate inflammation around implants and promote the repair phase [342]. However, an unwanted side effect of glucocorticoids on the surrounding tissue is the reduction of endogenous angiogenesis and the consequent wound healing delay [343,344]. The combined delivery of dexamethasone together with VEGF (a neo-angiogenetic growth factor) alone or with proportionally shared PDGF has been demonstrated to prevent the FBR, increase angiogenesis, and promote blood vessel maturation, respectively [345,346,347]. 

Besides VEGF and PDGF, a complex network of growth factors (EGF, FGF—fibroblast growth factor, GM-CSF granulocyte-macrophage colony stimulating factor, TGF-β) have been shown to control adhesion, migration, proliferation, and cell differentiation in wound healing (reviewed in [348]); therefore, biomaterials bearing these molecules can still show immunomodulatory properties. The subcutaneous administration of GW2580 (the inhibitor of CSF1receptor) has been shown to avoid the FBR to the alginate particle implants, by blocking the recruitment of innate and adaptive immune cells [349].

A striking anti-inflammatory effect on both acute and chronic inflammatory responses and a significant inhibition of foreign body giant cell and fibrous capsule formation was obtained using a low molecular weight superoxide dismutase mimic (a new class of drugs which imparts anti-inflammatory property to the material), covalently conjugated to a biomaterial [350]. A durable control of immune responses can be obtained by the loading of biomaterial surfaces with coatings containing NO [351], whose continuous and slow release results in reduced immune cell recruitment, probably due to the down-regulation of inflammatory cytokines, as well as the protein nitrosation [352]. Furthermore, NO may induce macrophages to a self NO production, thus explaining its long-lasting anti-inflammatory activity [353,354].

### 5.4. Immunomodulation by Integrins, Pro-Resolving Mediators, Cells, and Regulatory Pathways

Functionalization of surfaces with integrin binding sites [355] represents a powerful strategy in initiating distinct intracellular “outside-in” signal pathways mediating specific cell activation elicited by the recognition of integrin adhesion sites [356], thus preventing nonspecific cell–material interaction [357,358]. This technology will play an important role in the future development of biomaterials and scaffolds (reviewed in [359].

The local delivery of specialized pro-resolving mediators (SPMs, a group of endogenous molecules having a fundamental role in triggering signals ending the acute phase of the inflammatory response) [360,361] limiting both the recruitment of neutrophils and their ingestion by macrophages [362] shifted the macrophage phenotypic profile towards a M2 reparative response in vivo [363,364]. These mediators have already proved to be efficient at promoting wound healing [365], improving reepithelization and the formation of granulation tissue, as well as innervation [366], controlling the macrophage polarization induced after a chitosan scaffold implantation [364] in an obese diabetic mouse model.

Cell therapy methods, either by including immune cells as a possible reservoir/producer of a molecules inducer of specific biological events or by inducing their recruitment [1], are other possible strategies used to improve regenerative medicine systems. Thus, employing macrophages as a pro-angiogenic reservoir represents a possible chance of settling one of the basic problems in tissue engineering, i.e., the appropriate vascularization of thick engineered tissues [367]; encapsulated MSCs decreased the fibrotic response of the FBR compared to acellular hydrogels by down-modulating the classically activated macrophages [368] and conditioned medium obtained from macrophages induced by specific biomaterials was able to differentiate other cells [369]. 

Scaffold-encapsulated cells or 3D printing have gained interest in regenerative medicine since they represent new models of advanced fabrication techniques. These new bio-fabrication approaches mainly contribute to protecting the cells against stressful environmental events, such as exposure to ultraviolet light, reactive chemicals, and mechanical injury, but they also enhance their resistance against in vitro or in vivo perturbations due to inflammatory processes or immune cell interactions. It is documented that the encapsulation of cells limits their accessibility to antibodies, providing an immune-protective barrier [370,371,372], thus representing a promising tool to improve current adoptive T cell therapy strategies [373]. Moreover, it has been shown that the biomaterials (silk or hyaluronan-based) used to encapsulate cells are good systems for local delivery of cytokines [374] or for modulating macrophage polarization [375] by regulating cell-ECM interactions. Recently, it has been shown that the contemporary encapsulation of bone marrow stromal cells and macrophages in a matrix with defined stiffness can influence stromal cell fate both through direct-matrix-associated regulation and indirect macrophage-based modulation [376]. At the same time, encapsulation contributes to delaying clearance of the cells when transplanted in vivo, thus improving their therapeutic effects and protecting the host from the allogeneic transplantation of immune cells and graft versus host disease [377].

The previous examples have been mostly focused on structures/molecules that are characteristic of the immune response, but they are not exhaustive of the whole range of opportunities available for modulating interactions between implanted biomaterial and the receiving host.

Different strategies can intervene by acting on regulatory pathways upstream of protein production [378,379,380], by using Small interfering RNA (siRNA)-mediated gene silencing as a therapeutic approach to control the immune system and to reduce the detrimental effect of excessive inflammation during the tissue healing process [381,382,383,384] or by modulating miRNA signaling either by overexpression or inhibition [385,386,387,388,389,390,391].

## 6. Concluding Remarks and Open Questions

The immune system, for its central role in strategic regulatory processes, remains the most significant critical issue for the development of tissue engineering. On the one hand, the innate immune system capability to monitor, recognize, and clear foreign bodies activates a response that is unaware of the therapeutic potential of implanted biomaterials; on the other hand, biomaterial possesses characteristics that “irritate” the immune system. Although the inflammatory response is the first step of wound/tissue healing, it is also the underlying reason for the failure of many implanted scaffolds. The failure or the success of this match is determined by the ability of biomaterials to negotiate body sharing. 

Many immune cell subpopulations and immuno-modulating factors are involved in the different phases of healing and, despite advancing knowledge and innovative approaches, we are far from having clarified the complex mechanisms by which the immune system orchestrates various organs. In addition, even ongoing studies, targeted to investigate the impact of material properties on immune activation, have yet to fully elucidate the mechanisms through which this activation occurs.

It is important to achieve a detailed understanding of the innate immune inflammatory processes by which neutrophils and monocytes/macrophages can be activated by biomaterial surfaces in the absence of any specific cell surface receptor or cytosolic receptor signaling. 

The control of neutrophil mobilization and functions might be an interesting strategy to promote/modulate tissue regeneration. They are the first circulating cells of the innate immune response migrating after tissue injury, likely involved in macrophage polarization, but it is still unknown how exactly this happens. 

In addition, the reasons for which macrophages shift from an inflammatory into an anti-inflammatory phenotype in certain types of tissues, while a distinct population of anti-inflammatory macrophages is mobilized in others, are not clarified. Therefore, another key aspect could be to disclose the regulatory processes driving the increase of anti-inflammatory/anti-fibrotic macrophages in vivo and to further bring these mechanisms into regenerative strategies. Immune modulators administered through biomaterials and drug delivery systems promoting M2 (IL-4)-like phenotypes are currently being studied; however, it must be considered that M2-type macrophages are also involved in the development of fibrotic diseases.

The growing evidence that T cell subsets can have both anti-regenerative and pro-regenerative properties indicates these cells as possible cellular targets for intervention, possibly modulating T cell mobilization, activation, and conversion into Tregs.

The limited success of non-fouling coatings and surface functionalization methods, as well as the ECM biology still in its infancy, despite promising results of decellularized materials, together with the pioneering biomimetic strategies, represent further targets of future research in tissue engineering.

Moreover, 3D printing of tissue-engineered constructs is a new flexible system to create biomaterial-based scaffolds. This technique allows the custom “printing” of precise shapes and architectures, eventually encapsulating cells, but the elicited crosstalk between the host immune system and the printed materials has yet to be investigated. 

Another important variable not to be underestimated is age. If on the one hand, we can obtain answers from observing newborns whose macrophage populations display pro-regenerative capacities, on the other hand, with increasing frequency, tissue engineering approaches are considered among the therapeutic alternatives for diseases that instead occur more frequently in the elderly. Ageing is accompanied by phenotypic and functional changes of the immune system and by low-grade inflammatory activity reflected by increased circulating levels of pro-inflammatory cytokines. Epidemiological studies have suggested this chronic low-grade inflammation as related to several age-related diseases, sharing an inflammatory pathogenesis. Consequently, the baseline characteristics of immune cells and the surrounding immune environment might be different among these subjects and also different from those of young adult populations, thus adding a further biological variable to the study of the biomaterial design and to the chance of integration and tissue regeneration. However, despite the increasing use of implantable medical devices in aged patients, few studies are available that examine the effects of aging upon the host response to biomaterials and the implications of this response for long-term integration and function.

Increasing knowledge and awareness deriving from biological systems and new structural, chemical, and physical understandings of human-derived biomaterials, together with recent advances in synthesis technology, will open the way to new and more sophisticated biomaterial designs and prospects in the near scaffold technology. The future of this field will continue to grow and evolve with the collaborative development of tissue-engineered products that offer simple solutions to complex problems. Nevertheless, we should also be interested in knowing what the safety of immune-engineered biomaterials and their long-term efficacy will be.

## Figures and Tables

**Table 1:** Characteristics of synthetic and natural polymers.

Characteristics	Synthetic	Natural
Polymer Types	Poly(anhydride), Poly(propylene fumarate) (PPF), Poly(caprolactone) (PCL), Poly(phosphazene), Poly(lactic acid) (PLA), Poly(ether ether ketone) (PEEK) poly(glycolic acid) (PGA) poly(lactic-co-glycolic acid) (PLGA)	agarose alginate collagen fibrin, glycosaminoglycans hyaluronic acid, chitosan silk
Advantages	inert, high reproducibility, availability on demand, reduced costs, constant quality supporting industrial scale production, possibility to design or tune, mechanical properties, composition adaptable to needs, possibility to fabricate complex shapes, controlled degradation rate, long shelf life, cell attachment improvement, potential to deliver soluble molecules	readily available, mass producible, large quantities constantly available, cost, low immunogenicity, bioactive properties, binding sites for cells and adhesion molecules
Drawbacks	immune response, lower ability to interact with cells, strong inflammasome reaction	sterilization cost, in vivo source natural variability, lot-to-lot variability, limited mechanical properties, degradation rate difficult to control, unwanted immune reactions due to impurities
Host Innate Immune response	high	low
Host Adaptive Immune response	not applicable	low
Based on data from [2,3,4,5,8,19,141,142]		

**Table 2:** Surface chemistry: commonly explored chemical moieties.

Groups	-NH_2_ (Amino)	-OH (Hydroxyl)	-COOH (Carboxyl)	-CH_3_ (Methyl)
Surfaces	hydrophilic	hydrophilic	hydrophilic	hydrophobic
Charges	positive	neutral	negative	neutral
Focal adhesions	medium	high	medium	low
Ability to access fibronectin domains, integrin binding, cell adhesion	medium	high	medium	low
Inflammatory cell infiltration	high *(in vivo)*	high *(in vivo)*	low	high *(in vitro)*
Macrophage response	anti inflammatory	low inflammatory	inflammatory	
			low inflammatory	
			low inflammatory promoting regulatory T cell phenotypes *(mouse model)*	
Thickness of fibrotic capsules around the implant	high *(in vivo)*	high *(in vivo)*	low	high *(in vitro)*
Cell differentiation pathways	medium (osteoblasts)	high (osteoblasts)	medium (osteoblasts)	low (osteoblasts and myoblasts)

Based on data from [7,17,183,184,185,186,187,188,189,190,191,192,193,194].

**Table 3:** Biomaterial topography: size.

Size	Cell Types	Findings
Nano scale	Platelets	36 nm particles: induced activation and cell flattening 56 nm particles: decreased platelet activation
	Macrophages	smooth surfaces 50nm to 200nm nanodots: increased IL-6 secretion 50 nm nanodots: induced maximum cell spreading, focal adhesion, cell density
		reduced migration and activation on nanostructured titanium inhibition of iNOS (inducible nitric oxide synthase), NO (nitric oxide) and pro-inflammatory cytokines decreased migration on surfaces
	Dendritic cells	3 nm: enhanced activation, increased IL-12 and IFN-γ production increased pro-inflammatory T cell activity in co-culture 12 nm: increased IL-4 secretion skewed T cell immune responses toward wound healing
Nano- submicron scale	Macrophages	reduced initial adhesion on titanium surface less differentiated morphology reduced adhesion and pro-inflammatory cytokine release
Micron scale	Macrophages	micropatterning controlled cell shape stimulated cell elongation up-regulated M2 markers reduced inflammatory cytokine secretion protected cells from M1-inducing stimuli LPS (lipopolysaccharide) and IFN-γ
		2–40 μm particles: size-dependent production of IL-10 and TNF-α involved TLR-2 stimulation largest particles: did not induce cytokines
Meso scale	Macrophages	0.5 mm diameter particles: intraperitoneal fibrotic growth (mouse) 1.5–2 mm diameter particles: reduced fibrotic tissue formation in mice and non-human primates medium particles biased responses toward M1 inflammatory phenotypes larger particles caused a shift toward M2 immune regulatory and wound healing phenotypes

Based on data from [184,213,216,231,232,233,234,235,236].

**Table 4:** Biomaterial topography: shape.

Cell Types	Findings
Macrophages	internalization of gold nanorods was stronger compared to that of nanospheres owing to preferential uptake of the former via micropinocytosis
	the shape dependence of macrophage behavior was investigated by testing these cells with rods of varying lengths shorter rods were more rapidly internalized longer rods induced enhanced inflammatory mediators (IL-1α and TNF-α) since not readily phagocytosed
	the different shaped cross-sections of rods extruded from medical-grade materials affected the FBR extent: circular cross-sections induced the least-extensive reaction compared to pentagonal and triangular ones
	smooth surfaces led to less acute reactions than sharp; corners, acute angle surfaces
Neutrophils	the rough rather than smooth surface of polystyrene-polyethylene oxide particles boosted neutrophil recruitment and IL-1β production rough particles: preferentially taken up by macrophages, increased activation of inflammasome
Dendritic cells	titanium dioxide shaped as particles (diameters of 7–10 nm or 15–20 nm), or as nanotubes (diameters of 10–15 nm and lengths of 70–150 nm): induced shape dependent cytokine secretion, reactive-oxygen specie production, DCs maturation
	the shape dependence of DCs response was confirmed with antigen-coated gold spherical, rod-shaped or cubical nanostructures that elicited differential cytokine secretion and antibody production rod-shaped particles induced IL-1β, spherical and cubical ones induced TNF-α, leading to a less specific inflammatory response
T lymphocytes	collagen ECM scaffolds: critical role of Th2 cells in wound healing, induced a regenerative microenvironment supporting role of T CD8 and B cells

Based on data from [10,136,187,236,239,240,241,242,243,244].

## Abbreviations

aAPCs	artificial antigen presenting cells
Arg	arginase
Arg1	Arginase 1
CCL	C chemokine ligand
CD	cluster of differentiation
CH3	methyl
CO2	carbon dioxide
COOH	carboxyl
CR	complement receptor
CXCL	CX chemokine ligand
DAMPs	damage-associated molecular patterns
DCs	dendritic cells
ECM	extra-cellular matrix
EDC	1-ethyl-3-(3-dimethylaminopropyl) carbodiimide
FBGC	foreign body giant cell
FBR	foreign body reaction
GAG	glycosaminoglycans
IFN	interferon
Ig	immunoglobulin
IL	interleukin
iNOS	inducible nitric oxide synthase
lPS	lipopolysaccharide
MBVs	microvesicles
MHC	Major histocompatibility complex
miRNA	micro RiboNucleic Acid
NETs	neutrophil extracellular traps
NH2	amino
NO	nitric oxide
NOS	nitric oxide synthase
OH	hydroxyl
PAMPs	pathogen-associated molecular patterns
PCL	poly(caprolactone)
PDGF	platelet-derived growth factor
PEEK	poly(ether ether ketone)
PEG	poly(ethylene glycol)
PEO	poly(ethylene oxide)
PGA	poly(glycolic acid)
PLA	poly(lactic acid)
PLGA	poly(lactic-co-glycolic acid)
PPF	poly(propylene fumarate)
PRRs	pattern recognition receptors
ROS	reactive oxygen species
SF	silk fibroin
SIS	small intestine submucosa
SLA	sand-blasted, acid etched
TGF	transforming growth factor
Th	T helper
Ti	titanium
TLR	Toll-like receptors
TNF	tumor necrosis factor
VEGF	vascular endothelial growth factor
